# OUR1/OsbZIP1-*OsPIN9* regulatory module controls auxin-dependent crown root formation in rice

**DOI:** 10.3389/fpls.2025.1718647

**Published:** 2025-12-09

**Authors:** Yihao Dong, Cornelius Mbathi Wainaina, Takaaki Kojima, Yoshiaki Inukai

**Affiliations:** 1 Graduate School of Bioagricultural Sciences, Nagoya University, Nagoya, Japan; 2 Department of Horticulture and Food Security, Jomo Kenyatta University of Agriculture and Technology, Nairobi, Kenya; 3 Faculty of Agriculture, Meijo University, Nagoya, Japan; 4 International Center for Research and Education in Agriculture, Nagoya University, Nagoya, Japan

**Keywords:** rice, OsbZIP1 transcription factor, *OsPIN9*, auxin, crown root formation

## Abstract

Crown root (CR) development is a major determinant of rice root system architecture and influences nutrient uptake, tiller number, and grain yield. Although polar auxin transport mediated by PIN proteins is critical for CR formation, the transcriptional regulation of specific PIN members remains unclear. Here, we demonstrate a novel regulation of *OsPIN9* mediated by the bZIP transcription factor OUR1/OsbZIP1. Loss of *OUR1/OsbZIP1* function significantly promotes CR formation under nutrient-sufficient conditions, and this induction requires upregulation of the auxin efflux carrier *OsPIN9*, which we identify as a direct target of OUR1/OsbZIP1. Disruption of *OsPIN9* mostly abolished the enhanced CR phenotype of the *our1* mutant. Spatially adjacent patterns of upregulated *OsPIN9* and enhanced auxin signaling were accompanied by the induction of the CR initiation regulators *CRL1*, *CRL5*, and *OsWOX11*, defining an OUR1/OsbZIP1–*OsPIN9* module that integrates auxin transport and signaling to regulate CR development in rice.

## 1 Introduction

Rice is one of the most important cereal crops globally (Fukagawa and Ziska, 2019), serving as a staple food for more than half of the world’s population and providing nearly one-quarter of the global caloric intake (Fitzgerald et al., 2009; Muthayya et al., 2014; Mohidem et al., 2022). Root systems are central to plant growth, as they determine the amount of nutrients and water available, thereby directly impacting yield and underpinning agricultural productivity (Thorup-Kristensen and Kirkegaard, 2016; Lynch, 2019; Tracy et al., 2020, 2023; Holz et al., 2024). The rice root system consists of seminal roots (SR) and postembryonic shoot-borne crown roots (CRs; adventitious roots), both of which give rise to lateral roots (LRs) (Yamauchi et al., 1987; Rebouillat et al., 2009). Among these, CRs are the predominant components of the fibrous root system and play pivotal roles in anchorage, water and nutrient uptake and grain yield (Coudert et al., 2010; Mai et al., 2014; Meng et al., 2019; Wang et al., 2021).

CR formation proceeds through defined stages, from founder cell specification to primordium initiation, tissue patterning, vascular connections, and emergence (Itoh et al., 2005). Substantial evidence has established auxin as the central determinant of CR initiation: local auxin gradients at the stem base, notably the innermost ground meristem, where cell dedifferentiation and CR primordium (CRP) formation (Kitomi et al., 2008; Dabravolski and Isayenkov, 2025). Auxin gradients require polar auxin transport (PAT) mediated by the PIN-FORMED 1 (PIN1) efflux carrier and are maintained by endosomal recycling via GNOM (Benková et al., 2003); mutations in *CRL4*/*OsGNOM* result in impaired formation of the auxin gradient and inhibition of CR initiation (Geldner et al., 2003; Kitomi et al., 2008; Liu et al., 2009). Together with the co-expression of *OsPIN1b* and *CRL4* at the stem base, the defective PAT in *crl4* suggests that OsPIN1b-mediated PAT controlled by CRL4/OsGNOM is indispensable for CR initiation in rice (Xu et al., 2005; Kitomi et al., 2008).

Downstream of auxin transport, auxin perception by the TIR1/AFB SCF complex promotes AUX/IAA degradation, releasing ARF transcription factors to activate target genes (Gray et al., 2001; Tiwari et al., 2004; Dharmasiri et al., 2005; Chapman and Estelle, 2009). Among these targets, *CRL1*/*ARL1* (an *LBD* transcription factor) is indispensable for CR formation, as loss-of-function alleles exhibit severe CR initiation defects (Inukai et al., 2005; Liu et al., 2005). *CRL5* encodes an AP2/ERF transcription factor that acts downstream of auxin/ARF to promote CRP initiation while tempering cytokinin responses; *crl5* mutants produce fewer CRs. *CRL5* overexpression confers cytokinin resistance for CR initiation and promotes the type-A response regulators *OsRR1* and *OsRR2*. Notably, *crl1 crl5* double mutants exhibit additive phenotypes, indicating pathway complementarity (Kitomi et al., 2011). Although direct ARF occupancy at the *OsWOX11* promoter has not yet been demonstrated, *OsWOX11* functions as an auxin-inducible activator of CR development and *OsWOX11* transcripts accumulate at the stem base. *Oswox11* loss-of-function lines show markedly reduced CR initiation and delayed emergence, while *OsWOX11* overexpression drives precocious and ectopic CR formation (Zhao et al., 2009, 2015; Zhang et al., 2018; Geng et al., 2024).

Auxin activity during CR development is further tuned by regulatory modules that interact with cell-cycle control and tissue patterning. The miR156–*OsSPL3*–*OsMADS50* pathway modulates auxin signaling and transport components and reduces CR number when misregulated (Shao et al., 2019). Auxin–cytokinin crosstalk is integrated via OsWOX11 and ERF3 to promote CRP initiation and subsequent growth (Zhao et al., 2009, 2015), whereas *OsCKX4* is transcriptionally regulated by OsWOX11 and CRL1 fine-tunes cytokinin levels at CRP sites to maintain hormonal balance (Gao et al., 2014; Geng et al., 2023). Additionally, OsNAC2 acts as an upstream integrator of auxin–cytokinin signaling by directly binding to the promoters of *OsARF25* and *OsCKX4* (Mao et al., 2020). Collectively, these studies frame CR formation as an auxin-centered process dependent on coordinated transport, signaling, and hormonal crosstalk.

PIN proteins function as auxin efflux carriers and mediate PAT, thereby generating auxin gradients essential for root initiation and patterning (Friml et al., 2002, 2003; Vieten et al., 2005). The rice genome encodes at least 12 PIN family members, many of which exhibit distinct expression domains and functional specializations compared with their *Arabidopsis* counterparts (Wang et al., 2009; Miyashita et al., 2010). Expression analyses have shown that most *OsPIN* genes are active in the vascular tissues of the stem base (Wang et al., 2009), directing auxin flow toward the root tip via the mature vasculature, similar to the pattern reported in *Arabidopsis* (Benková et al., 2003). Functional studies have demonstrated that several *OsPIN* genes specifically contribute to CR development. For example, the knockdown of *OsPIN1* resulted in significant inhibition of CR emergence and development (Xu et al., 2005). Similarly, *OsPIN10a* regulates CR initiation, underscoring the importance of coordinated auxin efflux at the stem base (Wang et al., 2009; Zhang et al., 2012). *OsPIN9* is particularly notable as a monocot-specific *PIN* that is absent in dicots, suggesting a lineage-specific functional innovation (Paponov et al., 2005; Wang et al., 2009; Miyashita et al., 2010). More recently, *OsPIN9* was reported to be induced by ammonium and to mediate auxin redistribution in the basal internodes, thereby promoting CR initiation and enhancing tiller formation (Hou et al., 2021; Xu et al., 2022).

The basic leucine zipper (bZIP) transcription factor family is highly conserved in plants and integrates hormonal and environmental signals into developmental programs (Jakoby et al., 2002). In *Arabidopsis*, the bZIP protein HY5 is a central regulator of photomorphogenesis and has been linked to auxin transport and signaling (Cluis et al., 2004). Its rice homolog, OsbZIP1, regulates multiple traits, including flowering time, photomorphogenesis, nutrient uptake, root development and grain yield (Chai et al., 2021; Hasegawa et al., 2021; Bhatnagar et al., 2023; Tanaka et al., 2024; Xinli et al., 2024; Xiong et al., 2024). Under low nitrogen and phosphorus conditions, enhanced root length has been reported in *88n*, a loss-of-function mutant of *OsbZIP1*, along with improved yield, driven by increased Pi uptake and nitrogen use efficiency (Tanaka et al., 2024). The *vig1a* allele greatly enhanced seedling vigor, chilling tolerance, and grain production. The specific mutation of *vig1a* disrupts the interaction between OsbZIP1 and OsbZIP18, another HY5 homolog, suggesting that OsbZIP1 functions cooperatively with OsbZIP18 in diverse crucial biological processes that determine seedling establishment, chilling tolerance, and grain yield (Xiong et al., 2024). Notably, multiple *OsbZIP1* alleles are associated with robust root systems, suggesting that *OsbZIP1* exerts a conserved effect on root architecture (Hasegawa et al., 2021, 2022; Tanaka et al., 2024; Xiong et al., 2024). Consistent with this view, the loss-of-function *our1* allele regulates root development via altered auxin signaling (Hasegawa et al., 2021). However, the downstream targets of OUR1/OsbZIP1 during root development remain unclear.

To address this gap, this study investigated the mechanism by which OUR1/OsbZIP1 directly and negatively regulates *OsPIN9* to control CR development via an auxin-dependent pathway. Loss of *OUR1*/*OsbZIP1* function enhances CR formation under nutrient-sufficient conditions, at the stem base, upregulated *OsPIN9* expression increased auxin transport toward the innermost ground meristem, promoting auxin signaling and activating CR regulators. Genetic and molecular evidence establishes an OUR1/OsbZIP1–*OsPIN9* module that integrates auxin transport and signal activation to regulate CR formation in rice.

## 2 Material and methods

### 2.1 Construction of transgenic plants

The *pOsPIN9*-*OUR1cis* promoter-edited line and *OsPIN9* mutant were generated using the CRISPR/Cas9 system (Mikami et al., 2015). Guide RNAs targeting OUR1/OsbZIP1 binding site in *OsPIN9* promoter and *OsPIN9* exon 1 were designed using the CRISPOR-assisted website (Haeussler et al., 2016) and cloned into pZH_OsU6gRNA_MMCas9 vector (Mikami et al., 2015) to generate the *pOsPIN9*-*OUR1cis*-CRI and *OsPIN9*-CRI constructs, respectively. The *DR5:NLS-3×Venus* were constructed as previously described (Lucob-Agustin et al., 2020). To generate *pOsPIN9:NLS-3×Venus* construct, a 3 kb genomic fragment upstream of the *OsPIN9* translation start codon (ATG defined as +1) was PCR-amplified from wild-type ‘Kimmaze’ genomic DNA and cloned it into the pGWB1 vector (Nakagawa et al., 2007). To construct *p35s*:*OUR1*-*GFP*, the *OUR1*-*GFP* fragment was amplified from the *ProOUR1:OUR1-GFP* construct made in the previous study (Hasegawa et al., 2021), and cloned into the pENTR/D-TOPO^®^ vector and subsequently transferred into the pGWB502Ω vector using the Gateway LR reaction (Nakamura et al., 2009). All primers used in this study were listed in **Supplementary Table 1**.

The generated fusion constructs were introduced into the EHA 105 strain of *Agrobacterium tumefaciens* via electroporation. Subsequently, constructs were transformed into plants via *Agrobacterium*-mediated transformation (Hiei et al., 1994; Ozawa, 2009). Immature embryos harvested from 10 to 14 days after flowering were infected by *Agrobacterium* carrying the respective constructs. After 2 days of co-cultivation, infected immature embryos were transferred to a fresh resting medium containing 400 mg/L carbenicillin disodium salt (Nakarai, Kyoto, Japan) to remove *Agrobacterium*. Following this, Hygromycin-resistant calli were selected over 4 weeks on a selection medium containing 400 mg/L carbenicillin disodium salt and hygromycin 30 mg/L (Wako Pure Chemicals, Osaka, Japan). Proliferating calli were then transferred to a fresh pre-regeneration medium containing 200 mg/L carbenicillin disodium salt and hygromycin 40 mg/L. After 8 days of culture, these calli were transferred to a fresh regeneration medium containing 30 mg/L hygromycin B and cultured for 2 weeks.

### 2.2 Plant material and growth conditions

*Oryza sativa* cv. Kimmaze (KM) was the wild-type (WT) for the *our1* mutant (Hasegawa et al., 2021). The *pOsPIN9-OUR1cis* promoter-edited line was obtained by CRISPR/Cas9-mediated mutation of the OUR1/OsbZIP1 binding site cluster in the *OsPIN9* promoter in the WT background. The *pin9 our1* double mutant was generated by knocking out the *OsPIN9* gene in the *our1* mutant using the CRISPR/Cas9 system. *DR5:NLS-3×Venus* and *pOsPIN9:NLS-3×Venus* reporter constructs were introduced into the WT and *our1* mutants. Because KM is less amenable to *Agrobacterium*-mediated transformation, the *p35s:OUR1-GFP* construct was introduced into the transformable cultivar Taichung65 (Yara et al., 2001) for chromatin immunoprecipitation (ChIP) analysis.

Rice seeds were pre-germinated in water mixed with fungicide (0.25% [w/v] benomyl benlate; Sumitomo Chemical Co.) and placed in a growth chamber (MLR-351; Sanyo) at 28 °C with continuous light for 24 hours, followed by rinsing and soaking for 48 hours in tap water filtered using a water purifier (MX600; TORAY Industries) (Kawai et al., 2022b). Germinated seeds were transferred onto floating plastic nets in a 9-L black plastic box (32 cm height × 19 cm length × 19 cm width) filled with 10% nutrient solution (Colmer, 2003), which was replaced every 7 days. The full-strength nutrient solution contained (mol m^-^³): 3.95 K^+^, 1.50 Ca²^+^, 0.40 Mg²^+^, 0.625 NH_4_^+^, 4.375 NO_3_^-^, 1.90 SO_4_²^-^, 0.20 H_2_PO_4_^-^, 0.20 Na^+^, and 0.10 H_4_SiO_4_. Micronutrients (mmol m^-^³) were 50.0 Cl, 25.0 B, 2.0 Mn, 2.0 Zn, 1.0 Ni, 0.5 Cu, 0.5 Mo, and 50.0 Fe-EDTA. The solution also contained 2.5 mol m^-^³ MES, and the pH was adjusted to 6.5 with KOH, giving a final K^+^ concentration of 5.6 mol m^-^³. To assess nitrogen-form availability in the WT and *our1* mutant, we applied three adjusted nutrient solutions: complete (+Nut), ammonium-only (−NO_3_^-^), and nitrate-only (−NH_4_^+^). Solutions were replaced weekly and detailed compositions are provided in **Supplementary Table S2**.

To examine the responses to nutrient availability, germinated seedlings were first grown in filtered water for 7 days. The plants of each line were then divided into two groups: one group was continuously maintained in filtered water for 14 days and the other was transferred to a nutrient solution for 21 days. For the selected transgenic lines, seedlings were transferred to a 10% nutrient solution and cultivated in 9-L boxes with aeration to promote growth. The later-stage T_0_ plants were grown to maturity in a controlled greenhouse environment, and T_1_ seeds were harvested from individual T0 lines for subsequent analyses.

### 2.3 Electrophoresis mobility shift assay

The coding sequence of OUR1/OsbZIP1, codon-optimized for *Escherichia coli* usage, was synthesized (Eurofins, Japan) and cloned into the pMAL-c2 vector (New England Biolabs) for fusion with maltose-binding protein. The construct was introduced into *E. coli* BL21(DE3) and the recombinant proteins were expressed and purified according to the procedure described in a previous study (Kojima et al., 2010). To prepare DNA probes, 60 bp oligonucleotides were labeled with Cy5 fluorescent dye using a Klenow fragment (TaKaRa Bio, Japan) and purified on a column (NuclcoSpine Gel and PCR Clean-up, Macherey-Nagel, Germany) following the manufacturer’s instructions. DNA-binding reaction was performed at 4°C for 30 min in phosphate-buffered saline (PBS) (-) containing 1 mM 2-Mercaptoethanol, 25.3 nM probe, and 0.4, 0.6, 0.8 μM recombinant OUR1/OsbZIP1-DB proteins. The reaction mixtures were subjected to EMSA with 6% polyacrylamide gels in 0.5 x TBE buffer at 4°C. Cy5-labeled probes were analyzed using a Typhoon FLA9000 (GE Healthcare, USA) (Kawai et al., 2022a).

### 2.4 ChIP and qPCR analysis

ChIP was performed as previously described with minor modifications (Yamaguchi et al., 2014). Approximately 2 g of fresh root tissue from 4-day-old *p35s*: *OUR1*-*GFP* T_1_ seedlings was cross-linked with 1% (v/v) formaldehyde under vacuum for 10 min at room temperature, and the reaction was quenched with 0.125 M glycine for 5 min. The tissues were washed three times with cold PBS and ground to a fine powder in liquid nitrogen. Chromatin was isolated using lysis buffer [50 mM Tris-HCl (pH 8.0), 10 mM EDTA, 1% SDS, and protease inhibitor cocktail (Roche)] and sonicated to shear DNA to an average length of 200–500 bp using a Bioruptor sonicator (Diagenode) with 5 cycles of 10s on/30s off. After centrifugation at 13,000 × g for 10 min at 4°C, the supernatant was diluted 10-fold with ChIP dilution buffer [16.7 mM Tris-HCl (pH 8.0), 167 mM NaCl, 1.2 mM EDTA, 0.01% SDS, 1.1% Triton X-100] and precleared with Protein A/G agarose beads (Thermo Fisher Scientific) for 1 h at 4°C. Immunoprecipitation was performed with 5 μg anti-GFP antibody overnight at 4°C with gentle agitation. Antibody-bound chromatin complexes were pulled down using Protein A/G agarose beads for 2 h, followed by sequential washes with low-salt buffers, high-salt buffers, LiCl buffers, and TE buffers. Chromatin was eluted with elution buffer (1% SDS, 0.1 M NaHCO_3_) and reverse-crosslinked at 65°C overnight. DNA was purified using a PCR purification kit (Qiagen). qPCR was performed using SYBR Green master mix (Thermo Fisher Scientific) and specific primers targeting different sites in the promoter region of *OsPIN9* on the StepOnePlus Real-Time PCR system (Life Technologies). Fold enrichment was calculated relative to the input DNA using the % input method. The primer sequences are listed in **Supplementary Table 1**.

### 2.5 Expression analysis

Total RNA was extracted from the stem bases of the WT, *our1* mutant, and T_1_*pin9 our1* double-mutant seedlings using a NucleoSpin RNA Plant Kit (Macherey-Nagel, Germany), according to the manufacturer’s instructions. Quantitative real-time RT-PCR (qRT-PCR) was performed using the One-Step SYBR PrimeScript RT-PCR Kit II (Perfect Real Time; Takara Bio) and StepOnePlus Real-Time PCR (Life Technologies). The expression of each gene was normalized to that of *UBQ5* (*Os03g0234200*), which was used as an internal control. The primer sequences used for qRT-PCR are listed in **Supplementary Table 1**.

### 2.6 Measurement of root system architecture

The number of emerged CRs and tillers in the WT, *our1* mutant, and T_1_*pin9 our1* double mutant seedlings grown under nutrient-deficient or nutrient-sufficient conditions was manually counted at defined time points. To determine the number of CRPs, stem bases from WT, *our1*, and T_1_*pin9 our1* double mutants were embedded in 6% (w/v) INA agar and cross-sectioned into 150 μm-thick slices (DYK-1000N: Dosaka EM Co.). Sections were arranged sequentially, observed, and imaged with a Zeiss microscope using the Labscope software (Carl Zeiss, Oberkochen, Germany). Furthermore, to avoid double counting, CRPs that appeared in consecutive sections with overlapping positions and morphologies were considered as a single primordium.

### 2.7 Histological analysis

To observe DR5 reporter activity (*DR5:NLS-3×Venus*) and *OsPIN9* promoter activity (*pOsPIN9:NLS-3×Venus*) in the WT and *our1* mutants, stem bases were collected and fixed in 4% paraformaldehyde in PBS under vacuum for 1h, repeated twice. The samples were then washed with PBS for 30 min. The fixed samples were cross-sectioned into 150 μm-thick slices as described above and then cleared with TOMEI-I to visualize expression (Hasegawa et al., 2016). The sections were examined under a laser scanning microscope (FV3000; Olympus). Venus fluorescence was captured at an excitation wavelength of 488 nm and detected at 510–530 nm, whereas tissue autofluorescence was captured at an excitation wavelength of 405 nm and detected at 450–470 nm.

### 2.8 Statistical analysis

Differences in the morphological characteristics and gene expression between groups were compared using a two-tailed Student’s t-test or Tukey’s multiple-comparison test using GraphPad Prism.

## 3 Results

### 3.1 *our1* mutant shows enhanced CR formation under nutrient-sufficient conditions

**Figure 1:**
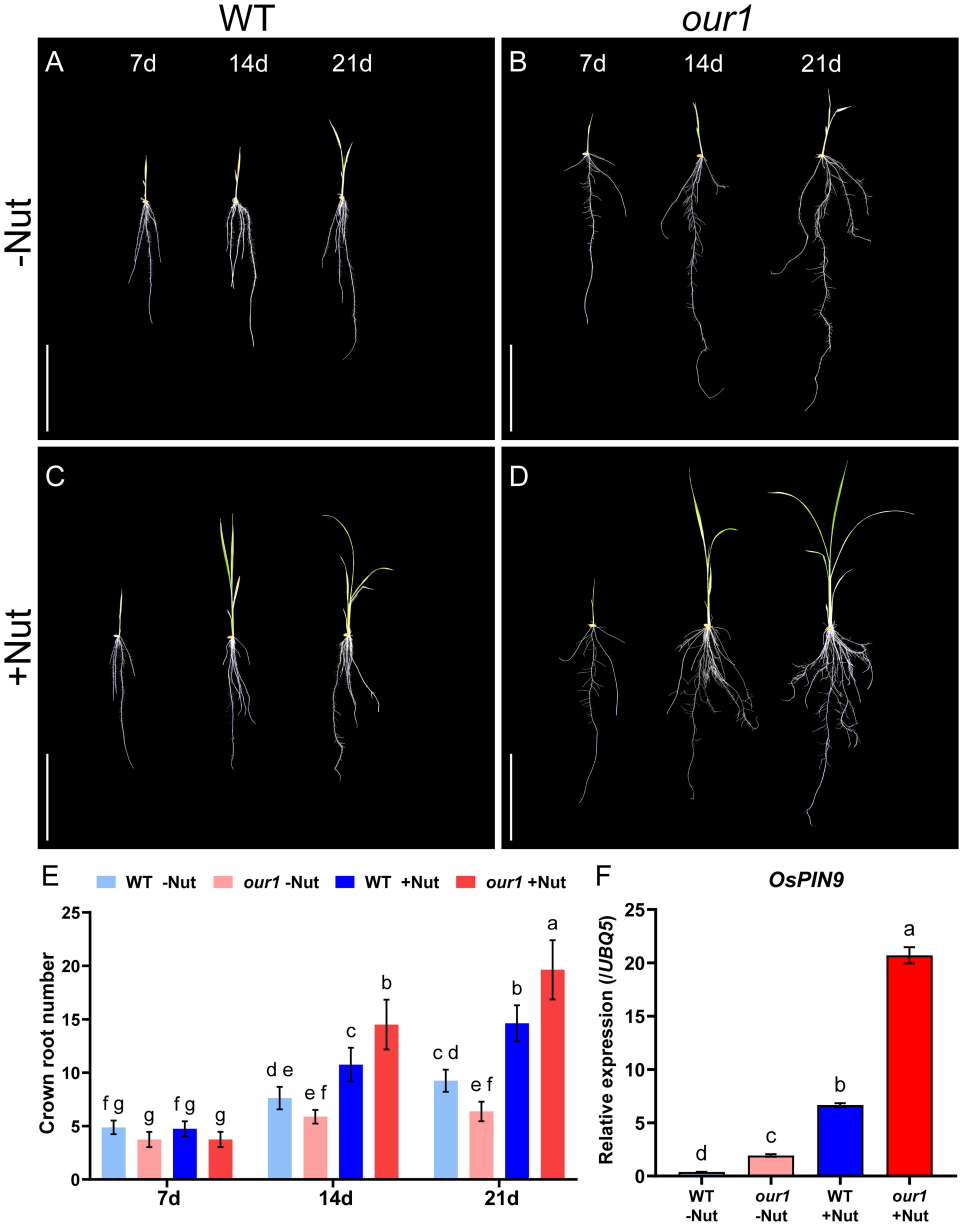
Enhanced CR formation in the *our1* mutant under nutrient-sufficient conditions. **(A, B)** -Nut (filtered water). 7-day-old seedlings were transferred to -Nut and imaged 7 and 14 days after transfer (14 and 21 DAS): WT **(A)** and *our1* mutant **(B)**. Scale bars = 10 cm. **(C, D)** +Nut (nutrient solution). 7-day-old seedlings were transferred to +Nut and imaged at 14 and 21 DAS as in **(A, B)**: WT **(C)** and *our1* mutant **(D)**. Scale bars = 10 cm. **(E)** Quantification of CR number corresponding to **(A–D)** for the WT and *our1* mutant under -Nut and +Nut conditions. Seedlings were transferred at 7 DAS; CR numbers were recorded at 7 DAS (pre-transfer), 14 DAS, and 21 DAS. Bars show mean ± SD (n = 8 plants per genotype per time point). Different lowercase letters indicate significant differences among groups (two-way ANOVA followed by Tukey’s multiple-comparison test, *P* < 0.05). **(F)** Relative expression of *OsPIN9* at the stem base of the WT and *our1* mutant under -Nut and +Nut conditions. Values represent mean ± SD (n = 3 technical replicates, repeated with two independent biological replicates). Different lowercase letters indicate significant differences among groups (one-way ANOVA followed by Tukey’s multiple-comparison test, *P* < 0.05). Primer sequences are listed in **Supplementary Table 1**.

**Figure 2:**
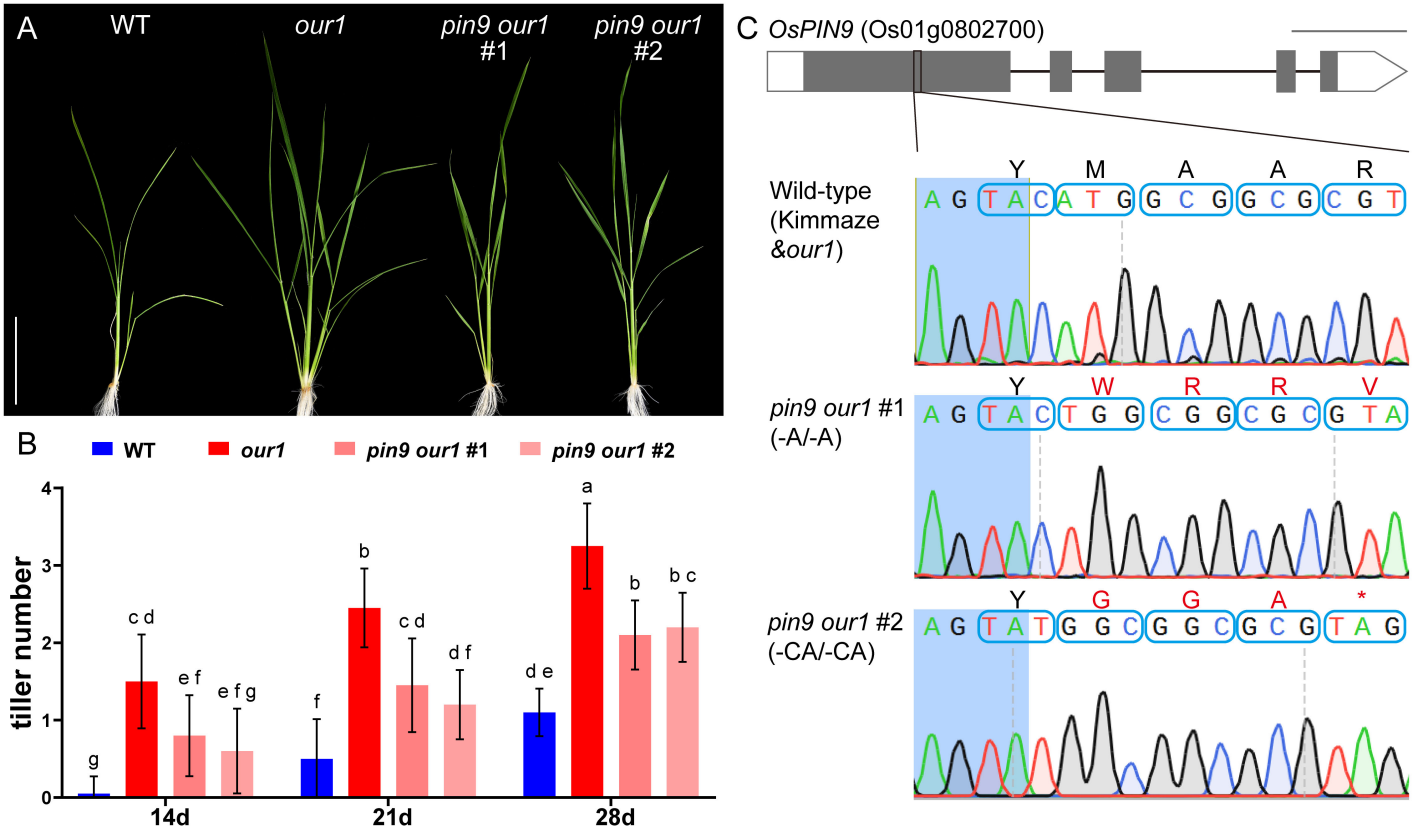
Tiller formation in the WT, *our1* mutant, and lines with different mutation of *OsPIN9* in the *our1* mutant. **(A)** WT, *our1*, and T_1_*pin9 our1* lines grown under +Nut conditions and imaged at 21DAS. Scale bar = 10 cm. **(B)** Tiller numbers of WT, *our1*, and T_1_*pin9 our1* lines counted at the 14, 21, 28 DAS under +Nut conditions. Bars show mean ± SD (n = 20 plants per genotype per time point, T_1_*pin9 our1* #2, n = 5). Different lowercase letters denote significant differences among genotypes (two-way ANOVA followed by Tukey’s multiple-comparison test, *P* < 0.05). Error bars were truncated at zero because tiller counts cannot be negative. **(C)** Genotypes of T_1_ lines with different mutations of *OsPIN9* in the *our1* mutant background. Structure of the *OsPIN9* gene (Os01g0802700). Gray and white boxes indicate exons and untranslated regions, respectively. Blue boxes display nucleotide triplets (codons), and the corresponding amino acids are shown above. Scale bars = 500 bp.

Consistent with a previous study of the root system suggesting that the *our1* mutation represses CR formation through restricting auxin signaling (Hasegawa et al., 2021), in filtered water, which is nutrient-deficient, both the WT and *our1* mutant exhibited only a modest increase in CR number as they grew, with the *our1* mutant consistently producing fewer CRs than the WT throughout the 21 days after sowing (**Figures 1A, B, E**). However, after 7-day-old seedlings were transferred to a nutrient solution, we found that the *our1* mutant exhibited a significantly enhanced CR number compared to the WT (**Figures 1C–E**). Likewise, tiller number increased in the *our1* mutant relative to that in the WT (**Figures 2A, B**). These results indicate the *our1* mutant is more responsive to nutrient availability, displaying strongly enhanced CR formation and increased tiller numbers under nutrient-sufficient conditions. Across nitrogen-form treatments, the *our1* mutant reached similarly high CR numbers in the complete nutrient solution supplying both NH_4_^+^ and NO_3_^-^ and the ammonium-only complete nutrient solution (NH_4_^+^ as the sole nitrogen source), whereas the nitrate-only complete nutrient solution (NO_3_^-^ as the sole nitrogen source), produced significantly fewer CRs than either condition, although still more than filtered water (**Supplementary Figure 1**). Taken together, these patterns suggest that ammonium availability is the principal driver of the enhanced CR formation in the *our1* mutant under nutrient-sufficient conditions.

### 3.2 *OsPIN9* is a potential contributor for enhanced CR formation in the *our1* mutant

CR initiation at the stem base requires a localized auxin maximum that is established by polar auxin transport (Kitomi et al., 2008; Dabravolski and Isayenkov, 2025), which is mainly regulated by PIN auxin efflux carriers (Friml et al., 2002, 2003; Vieten et al., 2005). *OsPIN9*, one of the PIN carriers, stands out as ammonium-inducible and its overexpression increased CR and tiller numbers (Hou et al., 2021). Because the phenotype of the *our1* mutant under nutrient-sufficient, especially ammonium-rich, conditions resembled that of *OsPIN9* overexpression lines (Hou et al., 2021), we hypothesized that *OsPIN9* is upregulated in the *our1* mutant and contributes to enhanced CR formation and increased tiller number. qRT-PCR analysis using the stem base revealed that the *our1* mutant exhibited significantly higher *OsPIN9* expression levels than the WT under both nutrient-deficient and -sufficient conditions (**Figure 1F**). These results suggested the repressive role of OUR1/OsbZIP1 on *OsPIN9* and support a model in which derepression of *OsPIN9*—amplified by nutrient stimulation—contributes to enhanced CR formation and increased tiller number in the *our1* mutant. A survey of *PIN* family expression in both WT and *our1* mutant showed that, besides the strongest upregulation of *OsPIN9*, *OsPIN8*, *OsPIN10a*, and *OsPIN10b* were modestly upregulated in the *our1* mutant, whereas most other members were unchanged or undetectable in this tissue (**Supplementary Figure 2**).

### 3.3 OUR1/OsbZIP1 binds to the *OsPIN9* promoter region

**Figure 3:**
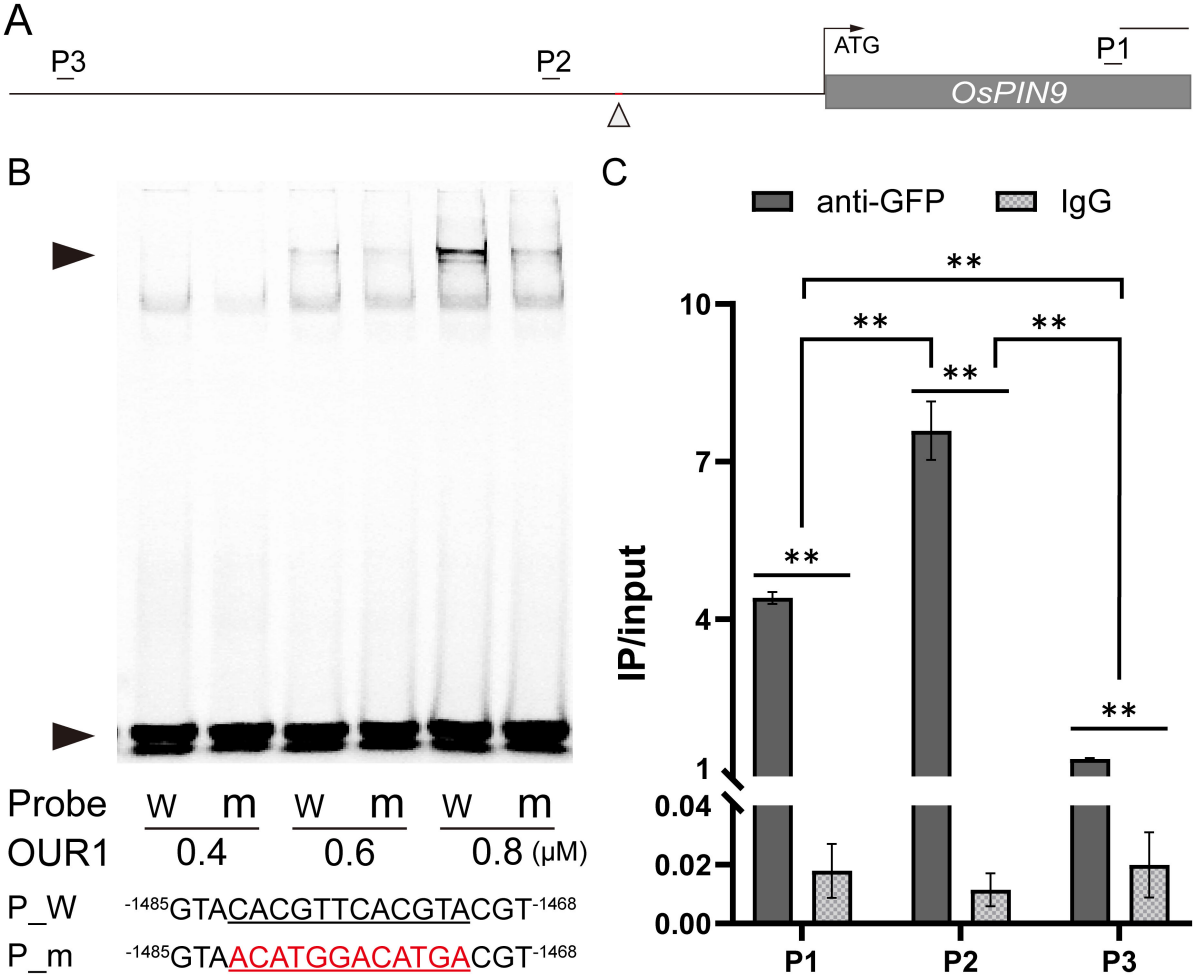
OUR1/OsbZIP1 binds to the *OsPIN9* promoter region. **(A)** Schematic of the *OsPIN9* locus, showing the upstream promoter, gene region (gray box). Upward arrowheads indicate the putative OUR1/OsbZIP1 binding site tested by electrophoresis mobility shift assay (EMSA), while short line mark primer sites used chromatin immunoprecipitation-qPCR (ChIP–qPCR) primer positions. Scale bar = 500 bp. **(B)** EMSA demonstrating binding of OUR1/OsbZIP1 to Cy5-labeled probes containing WT or mutated sequences in the *OsPIN9* promoter region. Upper and lower arrowheads indicate the shifted bands and free probes, respectively. (Lower) Probes sequences; underlined bases denote core motif, and red bases indicate mutations. Superscripted numbers refer to positions relative to the *OsPIN9* translation start site (+1). Full-length sequences of probes are shown in **Supplementary Table 1**. **(C)** ChIP–qPCR of the *OsPIN9* promoter in T_1_ seedlings in the WT background expressing *p35s*:*OUR1*-*GFP*. Cross-linked chromatin was immunoprecipitated with anti-GFP antibody. Normal IgG serves as a negative control. Values represent mean ± SD (n = 3 biological replicates), reported as the percentage of input DNA. Asterisks denote significant differences between genotypes (**P < 0.01; two-tailed Student’s t test). Primer sequences are listed in **Supplementary Table 1**.

The bZIP transcription factor OsbZIP1 preferentially binds to cis-elements containing an ACGT core sequence, such as the G-box (CACGTG), and related variants in the promoters of target genes (Izawa et al., 1993; Foster et al., 1994; Chai et al., 2021; Xiong et al., 2024). Because OsbZIP1 lacks a canonical activation domain and negatively regulates downstream targets (Bhatnagar et al., 2023; Chai et al., 2021; Xinli et al., 2024), we hypothesized that OUR1/OsbZIP1 repress *OsPIN9* by directly binding its promoter. In the *OsPIN9* promoter region, we identified a potential bZIP1 binding site consisting of two tandem motifs (CACGTTCACGTA) (**Figure 3A**). EMSA revealed that OUR1/OsbZIP1 bound to the probe containing the cis-element, as indicated by clear shifts at 0.6 and 0.8 μM protein concentrations, whereas binding was markedly reduced when the motif was mutated (**Figure 3B**). ChIP–qPCR showed significant enrichment of the *OsPIN9* promoter fragment near this site (**Figure 3C**), confirming OUR1/OsbZIP1 binding *in vivo*. In the promoter-edited line carrying a 25 bp deletion that disrupts the OUR1/OsbZIP1 binding site cluster within the *OsPIN9* promoter, *OsPIN9* transcript levels were significantly higher than in WT (**Supplementary Figure 3**), supporting that OUR1/OsbZIP1 acts as a repressor of *OsPIN9*. Taken together, these results demonstrate that OUR1/OsbZIP1 directly targets the *OsPIN9* promoter both *in vitro* and *in vivo*, thereby functioning as a negative regulator of *OsPIN9* transcription.

### 3.4 Upregulated *OsPIN9* is required for enhanced CR formation in the *our1* mutant

**Figure 4:**
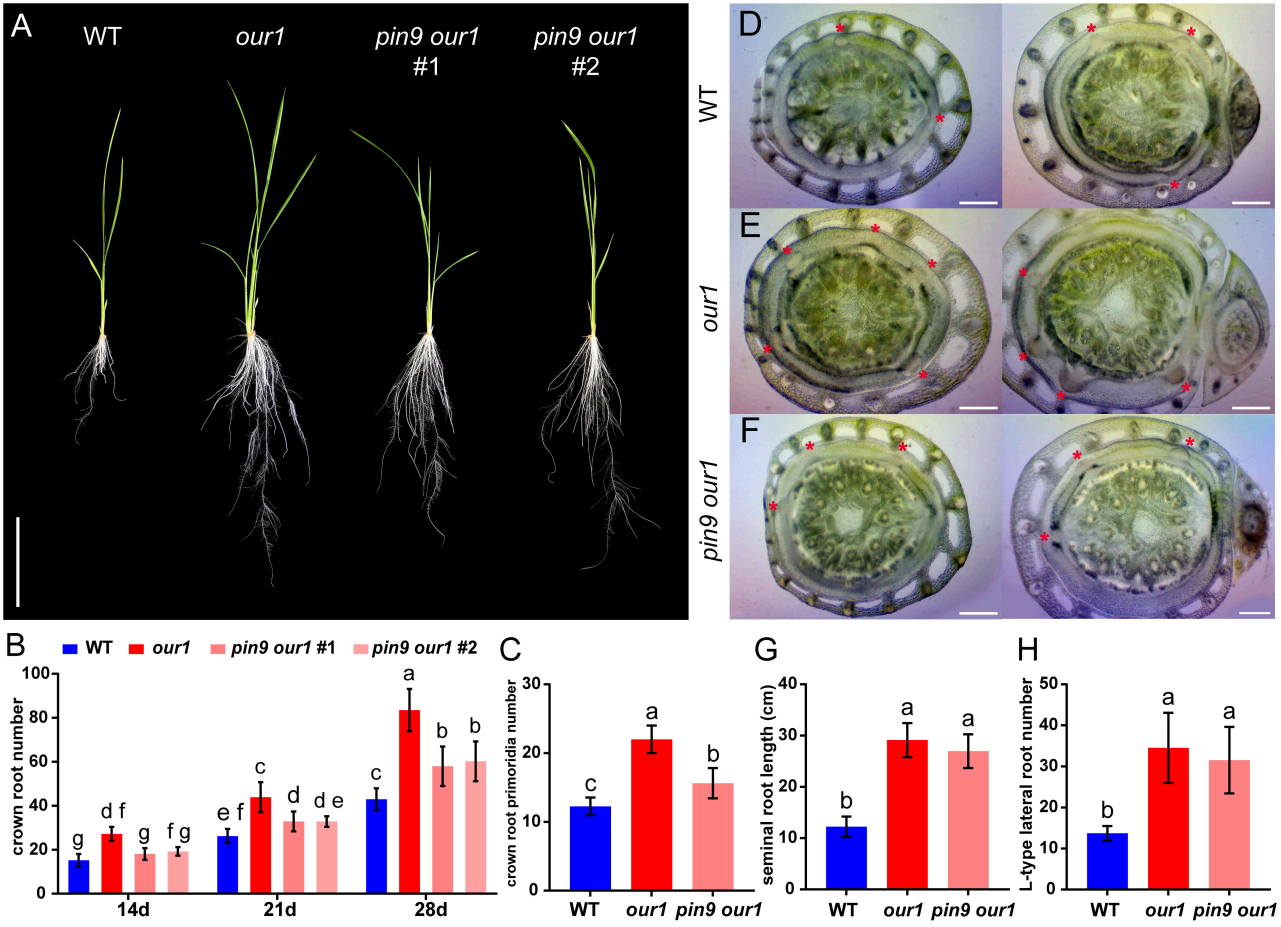
Upregulated *OsPIN9* promotes CR formation in the *our1* mutant. **(A)** 14-day-old seedlings of the WT, *our1* mutant, and T_1_*pin9 our1* lines grown under +Nut condition. Scale bar = 10 cm. **(B)** Time course of emerged CRs in the WT, *our1*, and T_1_*pin9 our1* lines grown under +Nut conditions at 14, 21, and 28 DAS. **(C)** CRP number of 14-day-old WT, *our1* mutant, and T_1_*pin9 our1* #1 grown under +Nut condition. **(D-F)** Cross sections of the stem base of the 14-day-old WT, *our1*, and T_1_*pin9 our1* double mutant #1 grown under +Nut conditions. Asterisks mark CRP. Scale bars = 0.5 mm. **(G, H)** seminal root length **(G)** and L-type lateral root number **(H)** of 14-day-old WT, *our1*, and T_1_*pin9 our1* #1 grown under +Nut condition. Data presentation, statistics, and sample sizes: Bars show mean ± SD. **(B)** Two-way ANOVA followed by Tukey’s multiple-comparison test. Different lowercase letters indicate significant differences among genotype–time groups (*P* < 0.05). **(C, G, H)** One-way ANOVA followed by Tukey’s multiple-comparison test; different lowercase letters indicate significant differences between genotypes (*P* < 0.05). Sample sizes: **(B)** n = 20 plants per genotype; T_1_*pin9 our1* #2, n = 5 **(C)** n = 8 per genotype; **(G, H)** n = 20 per genotype.

To confirm whether *OsPIN9* contributes to the enhanced CR phenotype of the *our1* mutant, we generated *OsPIN9* knockout lines in the *our1* mutant background using CRISPR/Cas9, hereafter referred to as *pin9 our1* double mutants (**Figure 2C**). Phenotypic analysis of T_1_ seedlings cultivated under nutrient-sufficient conditions revealed that the increased CR and tiller number in the *our1* mutant were significantly abolished in the *pin9 our1* double mutants (**Figures 2A, B**, **4A, B**), indicating that *OsPIN9* upregulation is required for enhanced CR formation. To confirm whether this effect was attributable to CR initiation, we examined stem base cross-sections to quantify CRP. Compared with the *our1* mutant, both the WT and *pin9 our1* double mutant displayed significantly lower CRP levels (**Figures 4C-F**), suggesting that *OsPIN9* promotes CR formation primarily by enhancing CRP initiation. Furthermore, the *pin9 our1* double mutant retained a modest increase in emerged CRs and CRPs compared to the WT (**Figures 4B, C**), suggesting the involvement of *OsPIN9*-independent pathways that contribute to the enhanced CR phenotype of the *our1* mutant.

Consistent with the previous characterization of the *our1* mutant, including elongated SR and increased L-type LR formation (Hasegawa et al., 2021), we investigated whether *OsPIN9* also underlies these traits. Despite *OsPIN9* being upregulated in the *our1* mutant even under nutrient-deficient conditions, loss of *OsPIN9* did not significantly affect SR length or L-type LR number in the *our1* mutant background (**Figures 4G, H**), indicating that upregulated *OsPIN9* in the *our1* mutant specifically induces CR formation but does not promote SR elongation or L-type LR formation.

### 3.5 Upregulated *OsPIN9* enhances auxin signaling at the stem base

**Figure 5:**
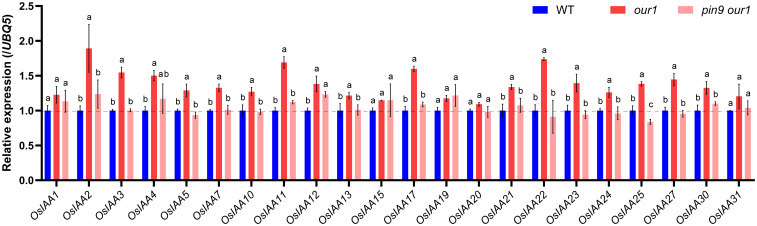
Expression of *AUX/IAA* genes at the stem base. Relative expression of 22 *AUX*/*IAA* genes at the stem base of the WT, *our1*, and T_1_*pin9 our1* #1 seedlings. Expression values were normalized to *UBQ5* (WT set to 1). Bars represent the mean ± SD (*n* = 3 biological replicates). For each gene, different lowercase letters indicate significant differences among genotypes (one-way ANOVA within genes, followed by Tukey’s multiple comparison test, *P* < 0.05). The primer sequences are listed in **Supplementary Table 1**.

Because *OsPIN9* encodes an auxin efflux carrier, we hypothesized that upregulated *OsPIN9* enhances auxin signaling at the stem base in the *our1* mutant. To test this hypothesis, we analyzed the expression of early auxin-responsive *Aux/IAA* genes at the stem base. Among the 31 *Aux/IAA* members in rice, 22 were selected based on their relatively high expression in the stems (Jain et al., 2006; Sato et al., 2013). Most genes were significantly upregulated in the *our1* mutant, with particularly strong increases in *OsIAA2*, *OsIAA11*, *OsIAA17*, *OsIAA22*, which returned to the near-WT baseline in the *pin9 our1* double mutant (**Figure 5**), indicating that enhanced auxin signaling in the *our1* mutant is largely mediated by *OsPIN9* upregulation.

**Figure 6:**
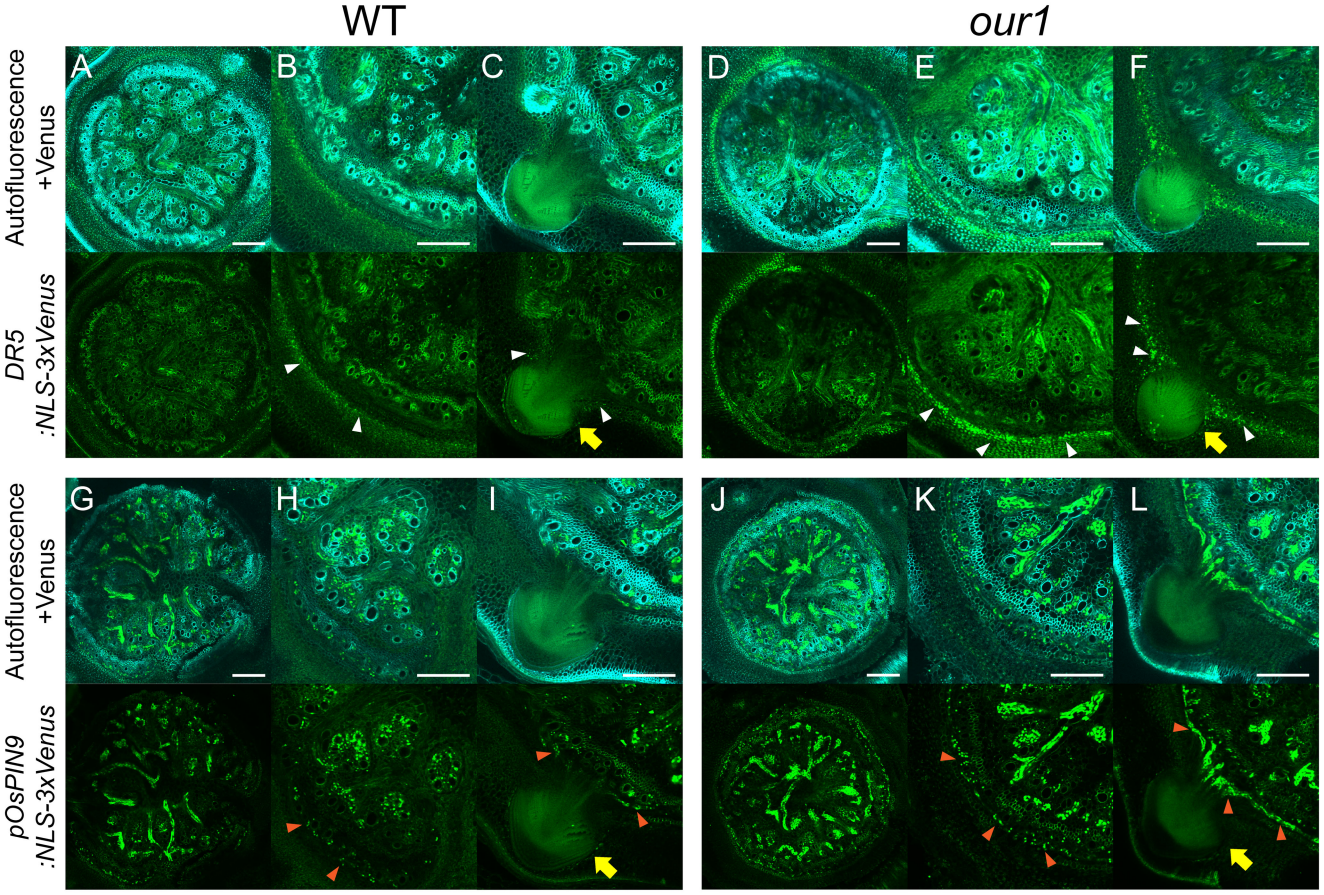
Patterns of auxin signaling and *OsPIN9* expression at the stem base. **(A–F)** Distribution of auxin signaling under +Nut conditions. Cross-sections expressing the *DR5*:*NLS*-*3×Venus* reporter are shown for WT **(A–C)** and *our1***(D–F)**. Close-up views showing regions without **(B, E)** and with CRP **(C, F)**. Venus fluorescence (green dots) denotes the DR5-driven auxin response, and autofluorescence is visible in the background. White arrowheads indicate localized auxin signaling, and yellow arrows indicate CRP. Scale bars = 200 µm. **(G–L)** Spatial pattern of *OsPIN9* promoter activity under +Nut conditions. Cross-sections expressing the *pOsPIN9*:*NLS*-*3×Venus* reporter are shown for WT **(G–I)** and *our1***(J–L)**. Close-up views show regions without CRP **(H, K)** or CRP **(I, L)**. Venus fluorescence (green dots) denotes *pOsPIN9*-driven signal, and autofluorescence is visible in the background. Orange arrowheads indicate localized *OsPIN9* expression and yellow arrows indicate CRP expression. Scale bars = 200 µm.

To further visualize how *OsPIN9* upregulation enhances auxin signaling at the stem base, we examined the activity of the synthetic auxin-responsive reporter *DR5:NLS-3×Venus* and the native *OsPIN9* promoter reporter (*pOsPIN9:NLS-3×Venus*) in stem base cross sections. Compared to the WT, DR5 reporter activity was strongly increased in the innermost ground meristem, where CRP initiation occurs in the *our1* mutant (**Figures 6A–F**). Consistently, *pOsPIN9* reporter lines showed upregulated *OsPIN9* expression in the peripheral cylinder of vascular bundles, which was spatially adjacent to the enhanced auxin signaling in the innermost ground meristem (**Figures 6G–L**). Together, these observations support a model in which *OsPIN9*-dependent efflux transports auxins from the peripheral cylinder of the vascular bundles to the ground meristem to further promote CRP initiation in the *our1* mutant.

### 3.6 Upregulated *OsPIN9* activates genes associated with CR formation

**Figure 7:**
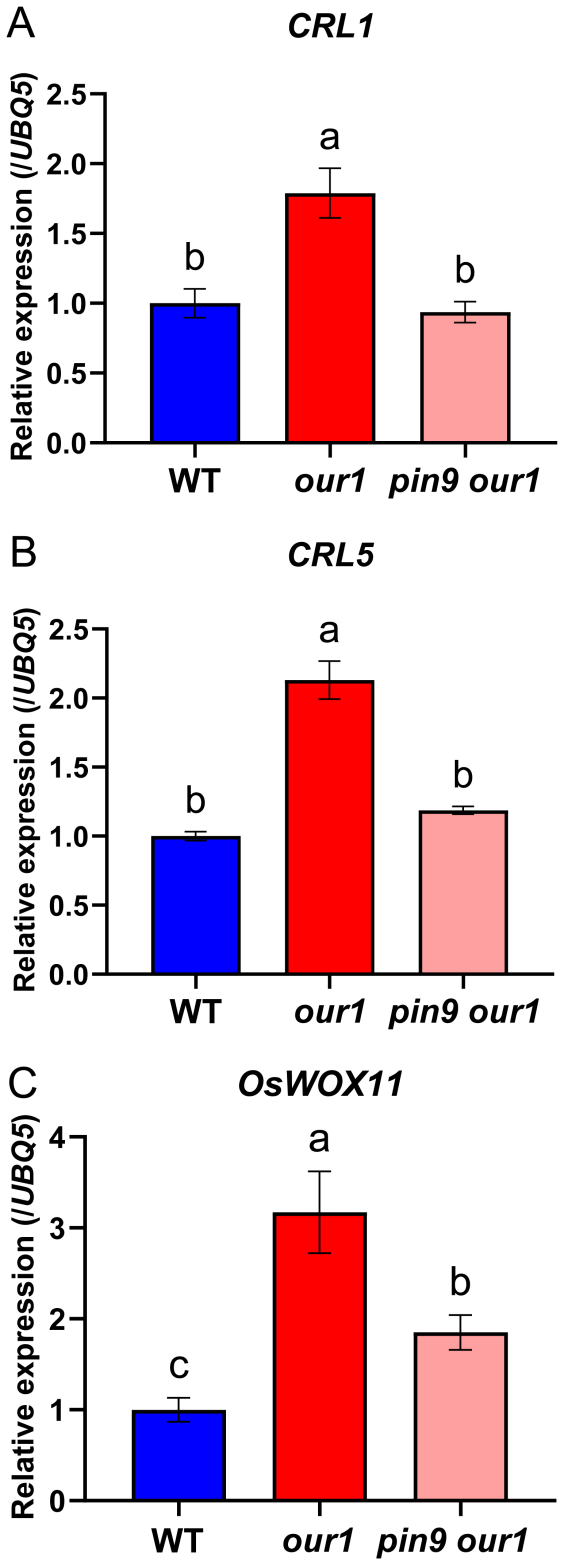
Auxin-responsive CR regulators are upregulated at the stem base in the *our1* mutant and depend on *OsPIN9*. **(A–C)** Relative expression levels of *CRL1***(A)**, *CRL5***(B)**, and *OsWOX11***(C)** at the stem base in the WT, *our1*, and T_1_*pin9 our1* double mutant #1 seedlings under +Nut conditions for 14d. Expression values were normalized to *UBQ5* (WT set to 1). Bars show mean ± SD (n = 3 technical replicates, repeated with two biological replicates with similar results). Different lowercase letters denote significant differences (one-way ANOVA with Tukey’s multiple-comparison test, *P* < 0.05). Primer sequences are listed in **Supplementary Table 1**.

Several auxin-inducible transcription factors, such as *CRL1*, *CRL5*, and *OsWOX11*, have been identified as key regulators of CR initiation and emergence (Inukai et al., 2005; Kitomi et al., 2011; Zhao et al., 2009; Zhang et al., 2018). Under filtered water, transcript levels of *CRL1*, *CRL5*, and *OsWOX11* were downregulated in the *our1* mutant than in WT at the stem base (**Supplementary Figure 4**), consistent with restricted auxin signaling in the *our1* mutant background (Hasegawa et al., 2021). Under nutrient solution, *CRL1*, *CRL5*, and *OsWOX11* were upregulated in the *our1* mutant compared to the WT, whereas their induction was markedly reduced in the *pin9 our1* double mutant (**Figures 7A–C**). Collectively, these results indicate that upregulated *OsPIN9* enhances auxin signaling at the stem base, leading to the activation of CR-related transcriptional regulators. Thus, the OUR1/OsbZIP1*–OsPIN9* regulatory module promotes CR formation in rice via an auxin-dependent pathway.

## 4 Discussion

In this study, we identified *OsPIN9* as a direct transcriptional target of OUR1/OsbZIP1 and established a module that regulates auxin transport and signaling at the stem base to promote CR development by initiating CRPs. Loss of OUR1/OsbZIP1 function derepresses *OsPIN9*, increasing auxin flux toward the CR initiation zone. Accordingly, the *our1* mutant showed markedly increased CR and tiller numbers under nutrient-sufficient conditions. EMSA and ChIP–qPCR confirmed the direct binding of OUR1/OsbZIP1 to the *OsPIN9* promoter, and disrupting the defined OUR1/OsbZIP1 binding site increases *OsPIN9* expression, supporting the negative regulation of *OsPIN9* by OUR1/OsbZIP1. Mechanistically, we propose that the upregulated *OsPIN9* enhances auxin efflux from the peripheral cylinder of vascular bundles toward the innermost ground meristem at the stem base, thereby triggering CRP initiation. Three lines of evidence support this model in *our1* mutant background: (i) increased DR5 reporter activity precisely at the CRP initiation site; (ii) upregulation of auxin-responsive *Aux*/*IAA* genes together with the CR inducers *CRL1*, *CRL5*, and *OsWOX11*; and (iii) loss or reduction of these responses, together with attenuation of the enhanced CR phenotype, in the *pin9 our1* double mutant compared to the *our1* single mutant. Taken together, these findings define an OUR1/OsbZIP1–*OsPIN9* module that regulates CR formation in rice via an auxin-dependent pathway.

The previous study showed that *our1/Osbzip1* mutant shows restricted auxin signaling, which promotes root elongation but suppresses CR formation (Hasegawa et al., 2021). By contrast, our data reveal a stem base specific increase in local auxin signaling that promotes CRP initiation in the *our1* mutant under nutrient-sufficient, especially ammonium-rich conditions. We propose that these opposite effects arise from different tissue and nutrient context. Consistent with auxin’s process-specific actions: high auxin level inhibits cell elongation in the root elongation zone (Sun et al., 2014, 2018; Nakamura et al., 2023), yet a local auxin maximum at the stem base triggers CRP initiation (Kitomi et al., 2008; Dabravolski and Isayenkov, 2025). According, under filtered water, globally restricted auxin signaling across the root system in the *our1* mutant promotes root elongation but suppresses CR formation. In this context, although *OsPIN9* is derepressed, the promotion of auxin signaling by *OsPIN9* is insufficient to induce CRP initiation, and CR numbers remain low in the *our1* mutant. By contrast, under nutrient-sufficient condition, it has been reported that other *PINs* such as *OsPIN1b* are upregulated and transport auxin from younger shoot part to the stem base (Sun et al., 2018). Therefore, under nutrient conditions, sufficient auxin is thought to accumulate at the stem base even in the *our1* mutant. The highly upregulated *OsPIN9* in the peripheral cylinder of vascular bundles is proposed to transport auxin into innermost ground meristem, establishing a local auxin maximum and thereby promoting CRP initiation in the *our1* mutant.

Previous studies have established that PIN-mediated polar auxin transport underpins key steps in CR development (Xu et al., 2005; Liu et al., 2009; Hou et al., 2021) and that *CRL1*, *CRL5*, and *OsWOX11* function as core auxin-responsive regulators of CR initiation and emergence (Inukai et al., 2005; Kitomi et al., 2011; Zhao et al., 2009; Zhang et al., 2018). Within the rice *PIN* family, *OsPIN9* is highly expressed in the peripheral vascular bundles of the basal internodes and has been implicated in CR development and tiller number, consistent with its role at the stem base (Wang et al., 2009; Miyashita et al., 2010; Hou et al., 2021). However, transcriptional regulation of *OsPIN9* remains unclear. Here, we newly identified a transcription factor OUR1/OsbZIP1, that directly represses *OsPIN9* expression. Together with previous evidence that OsWOX11 acts upstream of *OsPIN9* to promote *OsPIN9* expression and CR emergence (Zhou et al., 2017), a dual regulatory pathway was revealed: activation by OsWOX11 and repression by OUR1/OsbZIP1, controlling *OsPIN9* expression. Furthermore, because *OsWOX11* is auxin-inducible and *OsPIN9* controls polar auxin transport at the stem base, we propose that *OsPIN9*-driven auxin flux promotes *OsWOX11* expression, forming a potential modest positive feedback loop between *OsPIN9* and *OsWOX11* that promotes CR initiation. Nevertheless, under filtered water, *OsWOX11* expression is reduced in the *our1* mutant, consistent with the inhibited auxin signaling; thus, the proposed *OsPIN9*–auxin–*OsWOX11* positive feedback remains to be tested under conditions that support CR initiation. In addition to *OsPIN9*, *OsPIN8*, *OsPIN10a*, and *OsPIN10b* are upregulated in the *our1* mutant; notably, all three promoters contain ACGT-core bZIP motifs, suggesting potential regulation by OUR1/OsbZIP1. Among them, *OsPIN10a* has been functionally implicated in CR formation (Wang et al., 2009; Zhang et al., 2012). Further study will be required to determine whether these loci constitute a broader OUR1/OsbZIP1–*PIN* module contributing to CR initiation.

Previous studies demonstrated that *OsbZIP1* plays a critical role in regulating nutrient uptake, root system development, and grain yield (Hasegawa et al., 2021; Tanaka et al., 2024; Xiong et al., 2024). Our findings broaden this framework by identifying OsbZIP1 as an upstream integrator of auxin transport and signaling through the repression of *OsPIN9*, thereby extending its function to CR regulation. From an applied perspective, the OUR1/OsbZIP1–*OsPIN9* module provides a novel tractable point for optimizing rice root system architecture (RSA). The *our1* mutant has been reported to exhibit enhanced SR elongation and promoted LR formation, which maintains the yield under drought-prone conditions (Hasegawa et al., 2021, 2022). In parallel, *OsPIN9* overexpression has been associated with increased tiller number and reduced nitrogen fertilizer requirements (Hou et al., 2021), and *OsPIN9* expression is significantly induced by salt and drought treatments (Manna et al., 2022), together suggesting a strategy to enhance nutrient-use efficiency, abiotic stress adaptation, and productivity. These observations raise the testable prediction: *our1* mutant lines with activated *OsPIN9* activity may deliver yield gains under low nitrogen input and stress-prone conditions. To evaluate breeding utility, further comparative analyses across cultivars should be conducted to determine whether the OUR1/OsbZIP1–*OsPIN9* module can be generalized to design RSA that sustain yield under nutrient limitation and abiotic stress.

In conclusion, we identified OUR1/OsbZIP1 as a direct negative regulator of *OsPIN9*, thereby establishing an OUR1/OsbZIP1–*OsPIN9* module involving an auxin-dependent pathway to control CR formation in rice. This study provides a practical framework for manipulating CR development to improve RSA and crop performance.

## Data Availability

The raw data supporting the conclusions of this article will be made available by the authors, without undue reservation.
