# Supplementary material for: OUR1/OsbZIP1-OsPIN9 regulatory module controls auxin-dependent crown root formation in rice
Source: Front Plant Sci. 2025 Dec 9;16:1718647. doi: 10.3389/fpls.2025.1718647 (PMC12722469; doi:10.3389/fpls.2025.1718647)
Supplement: Supplementary file 1 [file DataSheet1.docx]

**Supplementary Figures**


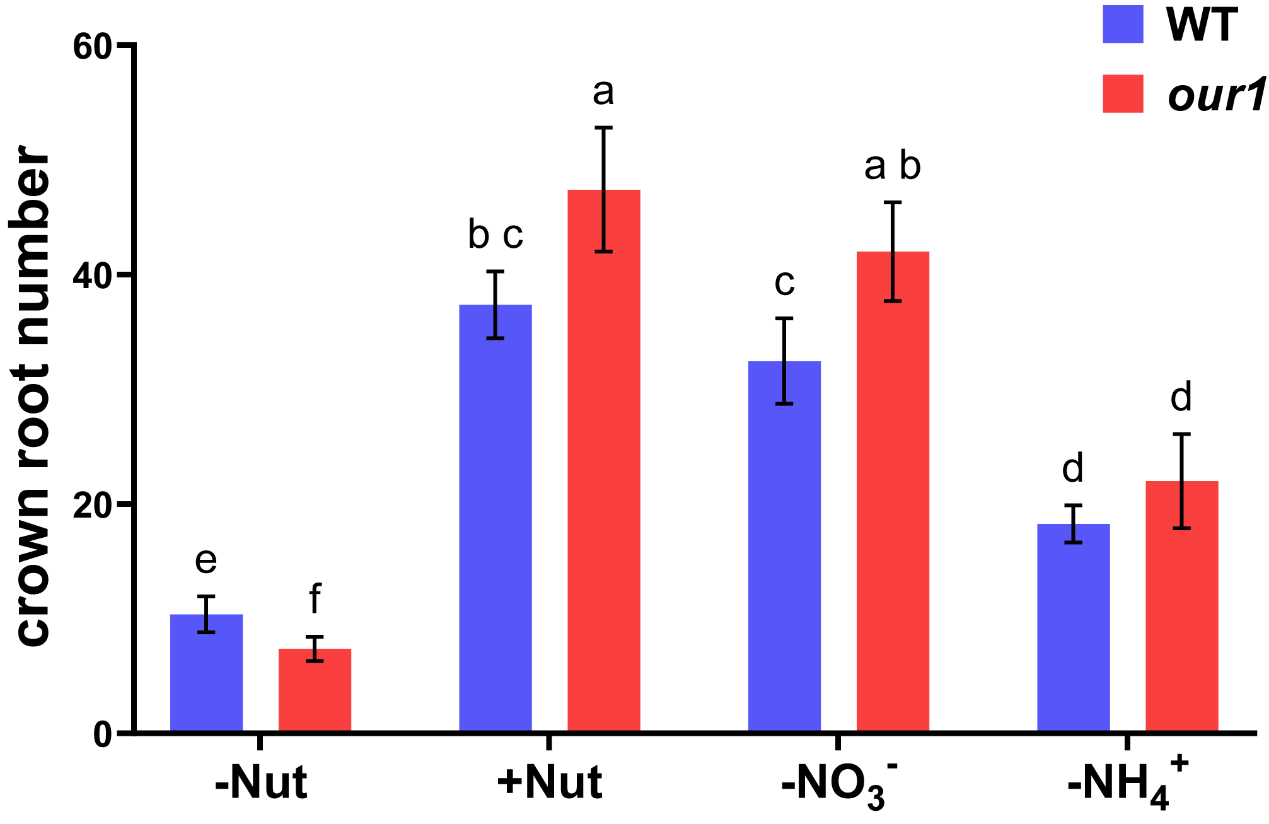


**FIGURE S1** **Crown root formation under filtered water and defined nitrogen-form nutrient solutions.** Numbers of emerged crown roots per plant in the wild-type (WT) and *our1* mutant lines grown for 21 DAS in filtered water (-Nut), complete nutrient solution (+Nut), ammonium-only complete nutrient solution (-NO₃⁻; NH₄⁺ as the sole nitrogen source), and nitrate-only complete nutrient solution (-NH₄⁺; NO₃⁻ as the sole nitrogen source). Bars show mean ± SD (n = 8 plants per genotype per condition). Different lowercase letters indicate significant differences among groups (two-way ANOVA with factors genotype and nitrogen form, followed by Tukey’s multiple-comparison test, *P* < 0.05).


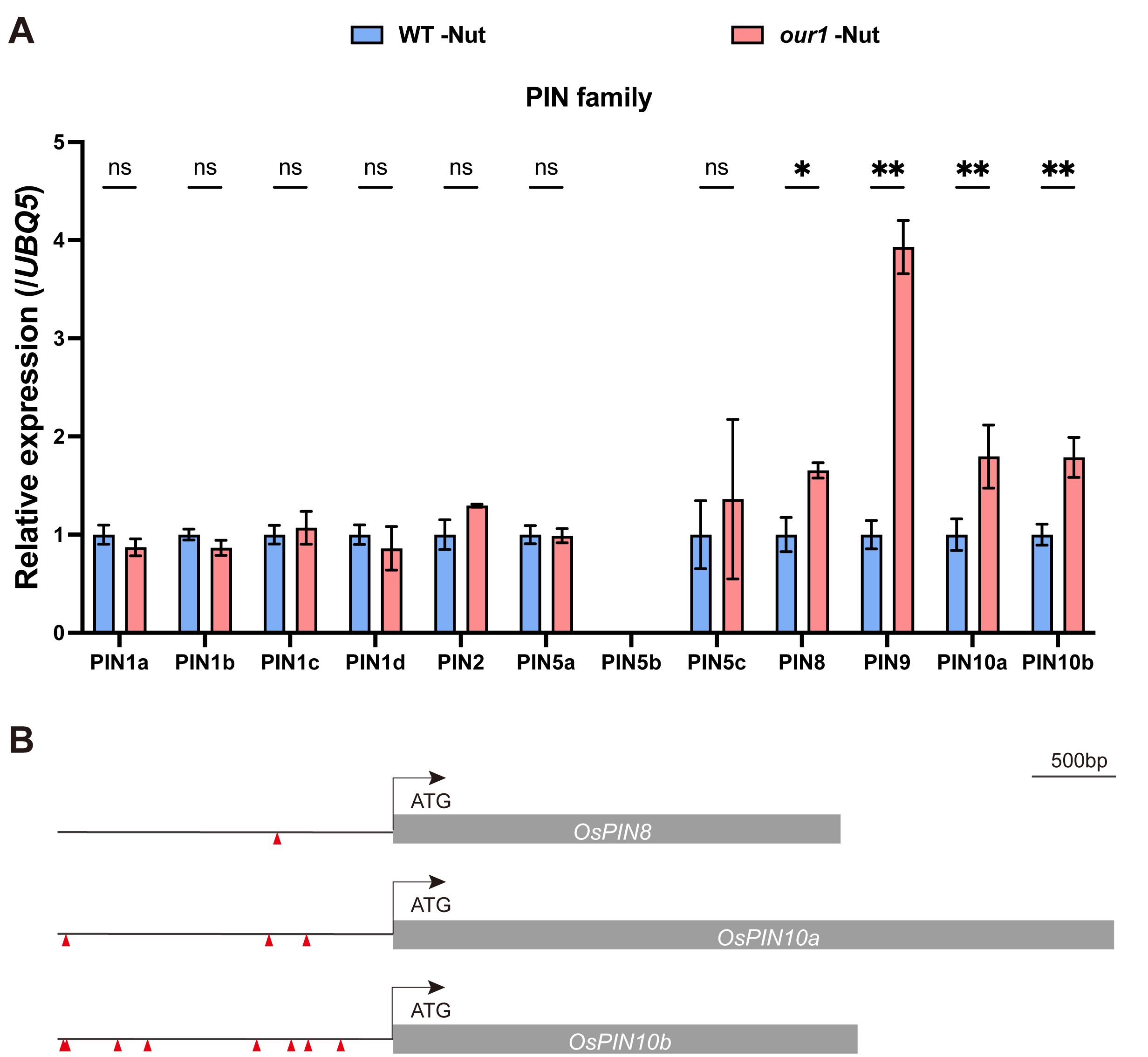


**FIGURE S2 Expression and promoter analysis of *OsPIN* family genes under filtered-water (-Nut) conditions.** **(A)** Relative expression of *PIN* family genes at the stem base of 14-day-old WT and *our1* mutant seedlings grown in filtered water (-Nut), normalized to *UBQ5* (*Os01g0328400*; WT set to 1). Bars represent mean ± SD (n = 3 technical replicates, repeated with two independent biological replicates). For each gene, significance was assessed by two-tailed unpaired Student’s t test and asterisks indicate significant differences between genotypes (^**^*P* < 0.01; ^*^*P* < 0.05; ns, *P* > 0.05). Transcripts of *OsPIN5b* were not detected in stem base of either genotype and therefore not shown. Primer sequences are listed in Supplementary Table 1. **(B)** Schematic of the *OsPIN8 (Os01g0764000), OsPIN10a (Os01g0643300), OsPIN10b (Os02g0743400)* loci, showing the 2-kb upstream promoter, gene region (gray boxes). Upward red arrowheads indicate potential binding sites for bZIP transcription factors containing the “ACGT” core motif. Scale bar = 500bp.


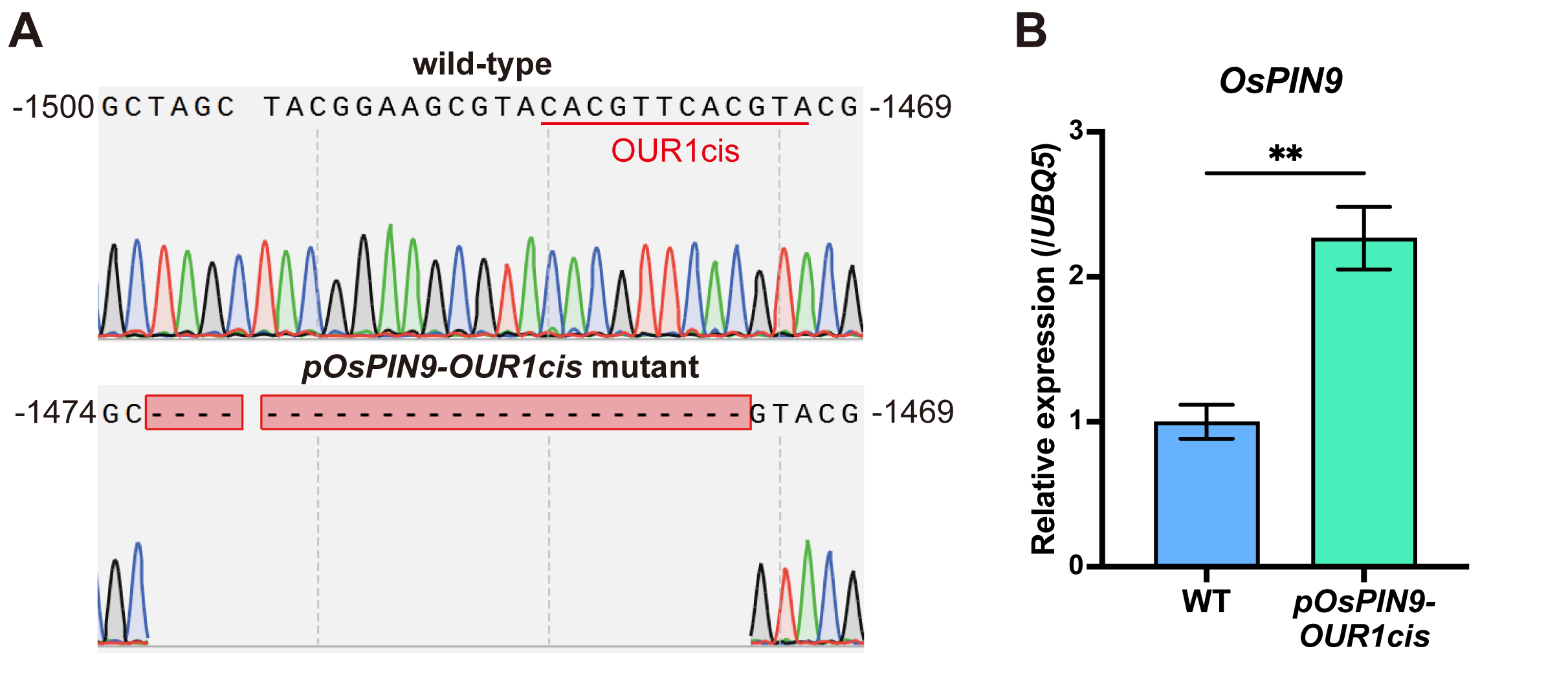


**FIGURE S3 Deletion of the OUR1cis site in the *OsPIN9* promoter increases *OsPIN9* expression. (A)** Sanger chromatograms of wild-type (WT) and the promoter-edited line (*pOsPIN9-OUR1cis*) around the OUR1/OsbZIP1 binding site in the *OsPIN9* *(Os01g0802700)* promoter. The predicted OUR1/OsbZIP1 cis-element is indicated by red underlines and refers as OUR1cis. The position numbers (bp) relative to the transcription start site are indicated at both ends. **(B)** Relative expression of *OsPIN9* at the stem base of 14-day-old WT and *pOsPIN9-OUR1cis* seedlings grown in filtered water (-Nut), normalized to *UBQ5* (*Os01g0328400*; WT set to 1). Bars represent mean ± SD (n = 3 technical replicates, repeated with two independent biological replicates). Significance was assessed by two-tailed unpaired Student’s t test and asterisks indicate significant differences between genotypes (^**^*P* < 0.01). Primer sequences are listed in Supplementary Table 1.


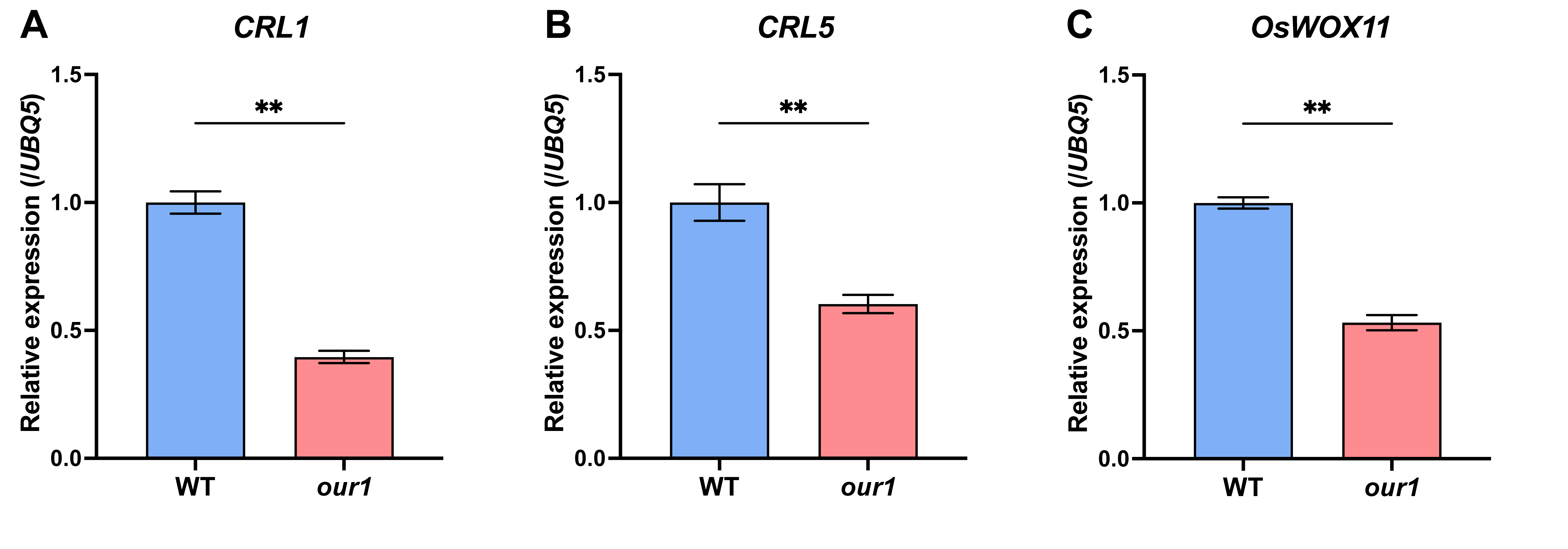


**FIGURE S4** **Auxin-responsive crown root regulators are downregulated at the stem base in the *our1* mutant under filtered water (-Nut). (A-C)** Relative expression levels of *CRL1* **(A)**, *CRL5* **(B)**, and *OsWOX11* **(C)** at the stem base in the 14-day-old wild-type (WT) and *our1* mutant seedlings grown in filtered water (-Nut). Expression values were normalized to normalized to *UBQ5* (*Os01g0328400*; WT set to 1). Bars show mean ± SD (n = 3 technical replicates, repeated with two biological replicates with similar results). For each gene, significance was assessed by two-tailed unpaired Student’s t test and asterisks indicate significant differences between genotypes (^**^*P* < 0.01). Primer sequences are listed in Supplementary Table 1.
